# 

**Published:** 1890-02-08

**Authors:** 


PRICE ONE PENNY.

No. 176, Vol. VII.—February 8th, 1890.

[Registered at the General Post Office as a Newspaper for transmission in the United Kingdom and Abroad.

WITH EXTRA NURSING SUPPLEMENT.
Per Annum, 6s. 6d., post free.
Monthly Parts, 6d.; post free 7½d.
Ditto, per Annum, 7s. 6d., post free.

